# Development and Validation of a Loop-Mediated Isothermal Amplification (LAMP) Assay for Rapid Detection of *Glaesserella* (*Haemophilus*) *parasuis*

**DOI:** 10.3390/microorganisms9010041

**Published:** 2020-12-25

**Authors:** Veronika Pilchová, Diana Seinige, Isabel Hennig-Pauka, Kathrin Büttner, Amir Abdulmawjood, Corinna Kehrenberg

**Affiliations:** 1 Research Center for Emerging Infections and Zoonoses, University of Veterinary Medicine Hannover, Foundation, 30559 Hannover, Germany; Veronika.Pilchova@tiho-hannover.de; 2 Lower Saxony State Office for Consumer Protection and Food Safety, 26203 Wardenburg, Germany; Diana.Seinige@gmx.de; 3 Field Station for Epidemiology in Bakum, University of Veterinary Medicine Hannover, Foundation, 30559 Hannover, Germany; Isabel.Hennig-Pauka@tiho-hannover.de; 4 Unit for Bioinformatics and Data Processing, Justus-Liebig-University Giessen, 35392 Giessen, Germany; Kathrin.Buettner@vetmed.uni-giessen.de; 5 Institute of Food Quality and Food Safety, University of Veterinary Medicine Hannover, Foundation, 30173 Hannover, Germany; Amir.Abdulmawjood@tiho-hannover.de; 6 Institute for Veterinary Food Science, Justus-Liebig-University Giessen, 35392 Giessen, Germany

**Keywords:** loop-mediated isothermal amplification (LAMP), *Glaesserella parasuis*, *Haemophilus parasuis*, pig, *infB* gene, rapid diagnostic, bronchoalveolar lavage fluid, BAL

## Abstract

*Glaesserella parasuis* is a fastidious pathogen that colonizes the respiratory tract of pigs and can lead to considerable economic losses in pig production. Therefore, a rapid detection assay for the pathogen, preferably applicable in the field, is important. In the current study, we developed a new and improved detection method using loop-mediated isothermal amplification (LAMP). This assay, which targets the *infB* gene, was tested on a collection of 60 field isolates of *G. parasuis* comprising 14 different serovars. In addition, 63 isolates from seven different closely related species of the family Pasteurellaceae, including *A. indolicus*, *A. porcinus*, and *A. minor*, and a species frequently found in the respiratory tract of pigs were used for exclusivity experiments. This assay showed an analytical specificity of 100% (both inclusivity and exclusivity) and an analytical sensitivity of 10 fg/µL. In further steps, 36 clinical samples were tested with the LAMP assay. An agreement of 77.1 (95% CI: 59.9, 89.6) and 91.4% (95% CI: 75.9, 98.2) to the culture-based and PCR results was achieved. The mean limit of detection for the spiked bronchoalveolar lavage fluid was 2.58 × 10^2^ CFU/mL. A colorimetric assay with visual detection by the naked eye was tested to provide an alternative method in the field and showed the same sensitivity as the fluorescence-based LAMP assay. Overall, the optimized LAMP assay represents a fast and reliable method and is suitable for detecting *G. parasuis* in the laboratory environment or in the field.

## 1. Introduction

*Glaesserella parasuis* (*G. parasuis*), previously named *Haemophilus parasuis*, is a small pleomorphic gram-negative fastidious bacterium belonging to the family Pasteurellaceae [1]. The pathogen colonizes the upper respiratory tract of healthy pigs early on in life. However, infection of an animal with *G. parasuis* can have many different effects depending on the type of the strain, health status of the herd, age of the pig, simultaneous coinfections, animal welfare conditions, and many other factors [2,3]. Besides being a commensal, *G. parasuis* is also the etiological agent of Glässer’s disease. This disease was described by Karl Glässer in 1906 and is characterized by polyserositis, arthritis, pericarditis, and serofibrinous inflammation of the pleura, which is often fatal [4]. It mainly affects weaners at the age of five or six weeks and specific pathogen-free (SPF) herds [5]. Acute or chronic progression of the disease mainly depends on the immunological status of the herd. In addition to Glässer´s disease, *G. parasuis* can also be secondarily involved in lung infections, e.g., after viral infections, cause acute pneumonia without polyserositis [6], or can be responsible for acute septicemia [7]. Although *G. parasuis* is mainly associated with domestic pigs, its presence in the nasal cavity of wild boars has been confirmed as well [8,9]. Overall, the pathogen causes considerable economic losses in the swine industry every year due to the necessary antibiotic treatment, vaccinations, and deaths of animals.

So far, 15 serovars of *G. parasuis* have been defined, each differing in virulence [10,11]. In addition, a relatively high prevalence of non-typable (NT) strains has been described [12]. Serovars 1, 5, 10, 12, 13, and 14 are recognized as highly virulent, causing high morbidity or mortality in SPF pigs within four days [4]. On the American continent, primarily serovars 5, 4, NT, 2, 12, 13, and 7 dominate in diseases [13,14], whereas in Europe, serovars 5, 4, 13, and NT [15,16] are dominant; in Asia, serovars 4, 5, 12, 13, NT, 14, and 2 are dominant [17,18,19].

*G. parasuis* is a commensal in the upper respiratory tract of pigs Amano, et al. [20]. Therefore, detection of the bacterium as a causative agent of Glässer´s disease should always be done in the affected tissues or body fluids, e.g., in lung tissue, bronchoalveolar lavage fluid, and joint fluid. The classical culture-based method is currently still the gold standard for detection, although it is very time-consuming and not always successful in detecting the pathogen. In fact, *G. parasuis* ranks among delicate and fastidious organisms and, consequently, bacterial cultivation may lead to false-negative results. *G. parasuis* could also be detected with several serological tests, such as immunohistochemistry [21] or immunodiffusion [22], but these methods are usually unsuitable for broad screenings with high sample quantities. In addition, antibodies may not be produced at early stages of infections. Further “gold standards” for detecting *G. parasuis* are PCR-based methods, e.g., conventional PCR [23,24], serotype-specific PCR [17], multiplex PCR [25], or real-time PCR [26]. These provide high sensitivity and specificity, but generally require costly equipment and are therefore unsuitable to be used directly in the field.

In 2000, a method named loop-mediated isothermal amplification (LAMP) was developed [27]. In principle, four specially designed primers recognize six distinct loci of the target DNA sequence in this LAMP assay. The method amplifies DNA with very high specificity and efficiency under isothermal conditions. A great advantage of the method is also the rapidity as a target sequence can be amplified in an hour. In addition, the device for the LAMP analyses is small and portable and so it can be used on site at any time. LAMP has become widely popular for detecting pathogens in food and clinical samples [28,29,30], even including the detection of SARS-CoV-2, the etiological agent of COVID-19 [31].

For detecting *G. parasuis*, LAMP has already been used in the first approaches [32,33,34]. In these previous studies, different target genes were used, but to date, neither an optimized temperature nor suitable primer concentrations are available. In addition, these studies used water baths and visual detection methods, making standardized interpretation of the results more difficult.

The aim of the present study was therefore to establish and validate a new improved LAMP assay for detecting *Glaesserella parasuis* in cultures but also in field samples. The validation process had to include the testing of DNA from reference and field strains, DNA from closely related bacteria, and also native samples.

## 2. Materials and Methods

### 2.1. Bacterial Strains

For this study, *G. parasuis* field isolates (*n* = 59) were used, which were collected on pig farms from different geographical regions in Germany during the years 2011–2013 [35]. Unfortunately, the serotype was only available for 29 of the 50 field isolates. In addition, *G. parasuis* type strain DSM 21448^T^ (Leibniz-Institute DSMZ, German Collection of Microorganisms and Cell Cultures GmbH, Brunswick, Germany) served as a positive control in every experiment. As negative controls for the validation experiments, bacterial species genetically closely related (88–90% identity of the target gene *infB*) to *G. parasuis* and frequently detected in the respiratory tract or infected joints of pigs were chosen. These strains included *Actinobacillus minor* CCUG 38923, *Actinobacillus indolicus* CCUG 39029, *Actinobacillus porcinus* CCUG 38924, and *Actinobacillus arthritidis* CCUG 24862 (all: Culture Collection University of Gothenburg (CCUG), Gothenburg, Sweden), as well as 11 *Mannheimia haemolytica*, 26 *Actinobacillus pleuropneumoniae*, 20 *Pasteurella multocida*, and two *Bordetella bronchiseptica* field isolates from the strain collection of the Institute for Veterinary Food Science, Justus-Liebig-University, Giessen, Germany. All *G. parasuis* isolates (*n* = 60) as well as all non-*Glaesserella* isolates (*n* = 62) tested in this study are listed in Table 1. 

### 2.2. Cultivation Conditions, Genomic DNA Isolation, and Species Confirmation

All isolates were cultured on chocolate agar (Carl Roth GmbH, Karlsruhe, Germany) supplemented with 10% defibrinated horse blood and were incubated at 35 °C for 24 to 48 h [36]. Genomic DNA of the bacterial strains and isolates was extracted with a Qiagen DNeasy Blood & Tissue Kit (Qiagen GmbH, Hilden, Germany) in accordance with the manufacturer´s instructions. Briefly, five to ten colonies of bacteria were harvested from agar plates with an inoculation loop and suspended into a mixture of 180 µL Buffer ATL, a tissue lysis buffer, and 20 µL Proteinase K. The lysis step was performed in a shaking heat block at 56 °C for 1.5 h. Subsequently, 200 µL Buffer AL and 200 µL 99.8% ethanol were added. After each step, the mixture was properly vortexed to improve the lysis process. The content of the tube was transferred into a DNeasy Mini spin column. Series of two washing steps with 500 µL of washing buffers were performed, followed by centrifugation steps (60 s, 6100× g). Finally, 100 µL Buffer AE was added as the final elution volume. For comparison, a rapid method was used in parallel for each clinical swab sample and DNA heat extraction was performed. For heat extraction, a volume of 100 µL of the transport medium was placed in a shaking heat block at 99 °C for 10 min at 400 rpm. Subsequently, the samples were centrifuged at 20,817× *g* for 5 min and the supernatant was transferred to a new 1.5 mL tube for further analysis. The results of both extraction methods were compared. To determine whether inhibition factors play a role in the LAMP assay, both undiluted and diluted DNA samples (1:10 in Buffer AE) were tested.

For species confirmation of *G. parasuis* isolates, a PCR assay targeting the 16S rRNA gene was performed, amplifying an 821 bp region as described earlier [24].

### 2.3. Native Samples and Culture-Based Detection of G. parasuis

For the investigation of clinical field samples, 36 swabs (MSwab^®^ Mast Group Ltd., Reinfeld, Germany) from different organs (pericardium, tonsils, bronchi, joints, serous membranes, and brain) were collected from August 2018 until September 2019 and were provided by the Field Station for Epidemiology in Bakum, University of Veterinary Medicine Hannover, Foundation, Germany. First, all samples were microbiologically examined there using routine diagnostic methods [37]. For this purpose, organs or swabs of the respective organs were streaked on Gassner agar (Oxoid Ltd., Hampshire, UK), Columbia Blood agar (Becton Dickinson, Heidelberg, Germany), Columbia CNA (colistin-nalidixic acid) agar (Becton Dickinson), and chocolate blood agar plates containing adenine dinucleotide (blood agar No. 2, Becton Dickinson). Gassner agar, Columbia blood agar and CNA agar plates were incubated aerobically for 16 h at 36 ± 1 °C. Chocolate agar plates were incubated under microaerophilic conditions with 8–10% CO_2_ for 16 h at 36 ± 1 °C. Colonies showing a colony morphology suspicious of *G. parasuis* were then subjected to biochemical tests (catalase, urease), tests for satellite phenomenon with *Staphylococcus aureus*, as well as X- and V-factor-dependent growth.

In addition, 36 tissue swab samples were transported in 1 mL Amies transport medium to the laboratory for LAMP analysis. There, DNA was extracted as described above.

### 2.4. Development of the LAMP Assay

#### 2.4.1. Target Gene and Primer Design

For the development of the LAMP assay, two different target genes, 16S rDNA and *infB,* which were described as suitable targets in previous studies [23,26], were taken into consideration. Three distinct primer sets, each consisting of six primers, were designed. For detecting the 16S ribosomal RNA gene, a 1477 bp nucleotide sequence deposited in the GenBank of the National Center for Biotechnology (NCBI), Bethesda, MD, USA (accession number AB078973.1), was used as target sequence. The primer set was developed by using the PrimerExplorer V4 software (Eiken Chemical Co. Ltd., Tokyo, Japan). The LAMP Designer (PREMIER Biosoft International ver. 1.15, Palo Alto, CA, USA) and Primer Explorer V4 software were also used to design two primer sets targeting the *infB* gene. The nucleotide sequence of the *infB* gene (1360 bp) was available from the National Center for Biotechnology (NCBI) GenBank (Bethesda MD, USA) under accession number DQ410886. All the initially used primers are shown in supplementary Table S1.

**Figure 1:**
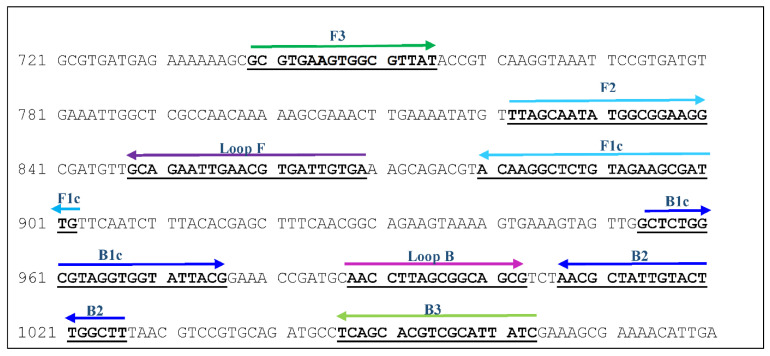
Nucleotide sequence of the *infB* gene (accession no. DQ410886) with binding positions of the LAMP primers.

By using the Basic Local Alignment Search Tool (BLAST) (https://blast.ncbi.nlm.nih.gov/Blast.cgi), the target sequences were compared to sequences of other bacterial species in the databases. The sequences from the species with the highest identity at the nucleotide sequence level (*Actinobacillus minor*, accession number EF055562.1, 90% identity; *Actinobacillus indolicus*, EF059971.1, 89% identity; *Actinobacillus porcinus*, EF059970.1, 88% identity; *Actinobacillus porcitonsillarum*, EF055563.1, 88% identity) were selected and matched with the sequence of the *infB* gene from *G. parasuis.* In this way, the species-specific nucleotide regions of *G. parasuis* were identified and their location was used for the final selection of the primer set. The chosen primer set (Table 2) was synthesized by Eurofins Genomics (Eurofins Genomics GmbH, Ebersberg, Germany) and consisted of six individual primers—two inner primers, two outer primers, and two loop primers (Figure 1). The forward inner primer (FIP) consisted of the F1c and F2 sequences, the backward inner primer (BIP)—of the B1c and B2 sequences. F3 and B3 primers represented the outer primers.

Since it was shown in previous studies that increasing the primer concentrations of FIP, BIP, loop F, and loop B (not F3 and B3) accelerates the amplification process [38], the primers were used in different concentrations (Table 3).

#### 2.4.2. LAMP Reaction Mixture and Amplification

**Figure 2:**
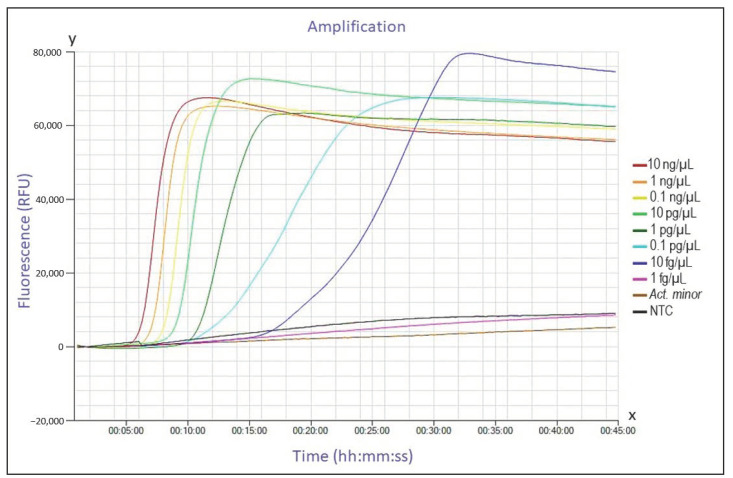
Example of a typical LAMP run in Genie^®^ II. The graph was created in the program Genie Explorer and shows an analytical sensitivity test using the concentrated master mix. The x-axis shows the amplification time (hh:mm:ss), while the y-axis shows the fluorescence expressed in relative fluorescence units (RFU). Red, orange, yellow, green, dark green, blue, dark blue, and pink are the colors used to represent ten-fold serial dilutions of *G.* (*H.) parasuis* (DSM 21448) genomic DNA from 10 ng/µL to 1 fg/µL, respectively. The brown curve represents DNA from *Actinobacillus minor* strain CCUG 38923^T^ (0.1 ng/µL) and the black curve represents the no template control (NTC).

Each tube with a total of 25 µL reaction mixture contained 15 µL Isothermal Master Mix ISO-001 (OptiGene Limited, Horsham, UK), 2.5 µL primer mix, 2.5 µL nuclease-free water, and 5 µL DNA template. The master mix included DNA polymerase, thermostable inorganic pyrophosphatase, reaction buffer, MgSO_4_, deoxyribonucleotide triphosphates (dNTPs), and double-stranded DNA (ds-DNA) binding dye. A mixture made of master mix, primer mix, and nuclease-free water served as the no template control in each run. The DNA concentrations of all positive and negative controls were quantified with the Nanodrop 2000c (Thermo Fisher Scientific Inc., Waltham, MA, USA) and subsequently adjusted to 0.1 ng/µL. For amplification, a Genie II^®^ (OptiGene Limited, Horsham, UK) instrument was used along with Genie^®^ strips—proprietary 8-microtube strips. Genie II^®^ is a portable device consisting of two separate heating blocks, each for one strip, and a TFT/LCD display (thin film transistor/ liquid crystal display) to observe the reaction in real time. All data from the Genie^®^ II device were processed in the software program Genie Explorer available at http://www.optigene.co.uk/support/. After preparing the reaction mixture, the solution was pipetted into tubes and incubated under isothermal conditions. At the end of the amplification, a melting step was performed beginning at 98 °C and decreasing 1 °C per minute to an end point of 80 °C. Results were evaluated in the Genie Explorer program as represented characteristically in Figure 2. Optimization of the reaction temperature was performed with a temperature gradient on a series of seven identical DNA samples from positive control strain *G.* (*H.) parasuis* DSM 21448 concentrated at 0.1 ng/µL. The temperature gradient ranged from 63 °C to 69 °C in steps of 1 °C.

### 2.5. Alternative LAMP Assay with Colorimetric Detection and Gel Electrophoresis

**Figure 3:**
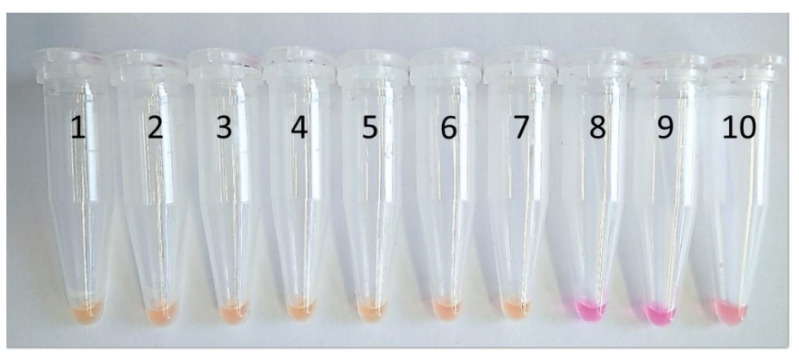
Results of the WarmStart Colorimetric LAMP 2X Master Mix assay for detection of LAMP amplicons with the naked eye. Samples 1–8: serial dilutions of DNA from strain *G.* (*H.) parasuis* DSM 21448 starting at concentrations of 10 ng/µL up to 1 fg/µL. Sample 9: DNA from *Actinobacillus minor* CCUG 38923^T^. Sample 10: no template control.

In addition, an alternative LAMP assay with visual amplicon detection based on a color change was tested. The isothermal OptiGene Isothermal Master Mix was replaced by the colorimetric WarmStart LAMP 2X Master Mix (New England BioLabs, Frankfurt am Main, Germany). This Master Mix contains a pH indicator, phenol red, in a low-buffer reaction solution, which causes a color change from pink to yellow depending on the amplification process. This assay was tested to provide an alternative for a field application as given by visualization of the process with the naked eye. This can speed up and simplify the screening process in the field. The same serial dilution steps used in the Genie II^®^ amplifications (analytical sensitivity test) were placed in a heating block at 66 °C for 45 min when using this alternative assay (Figure 3). To confirm the reaction of the colorimetric master mix, detection of the LAMP amplicons was performed by gel electrophoresis. An amount of 6 µL of the amplified product (ten-fold serial dilution from 10 ng/µL to 1 fg/µL) and a 1 kb Plus DNA ladder (Thermo Fisher Scientific) were transferred onto a 2% agarose gel with a running time of 90 min at 200 V. The final product was stained with ethidium bromide and was subsequently made visible under UV light.

### 2.6. Analytical Sensitivity of the LAMP Assay

The analytical sensitivity was evaluated based on a ten-fold serial dilution of nucleic acid extracts from the reference strain *G. parasuis* DSM 21448^T^ in TE (Tris EDTA) buffer. The test concentrations ranged between 10 ng/µL and 1 fg/µL. Two primer concentration variants—standard and concentrated—were tested (Table 4). Three independent experiments were performed.

### 2.7. Analytical Specificity of the LAMP Assay

The concept of analytical specificity is often described by inclusivity defined according to the Association of Official Analytical Chemists (AOAC) as “strains or isolates or variants of the target agent that the method can detect” (selectivity) and exclusivity—“strains or isolates or variants of the target agent that the method must not detect” (cross-reactivity) [39]. A total of 43 strains of *Glaesserella parasuis* with 14 different serotypes were used for inclusivity testing [35]. A total of 29 *Actinobacillus* spp. isolates, 11 *Mannheimia haemolytica* isolates, 20 *Pastereulla multocida* isolates, and 2 *Bordetella bronchiseptica* isolates were used for the tests of exclusivity (the isolates used are shown in Table 1).

### 2.8. Determination of the Limit of Detection

To investigate the limit of detection, a suspension of *G. parasuis* DSM 21448^T^ in Müller–Hinton II bouillon enriched with chicken serum and NADH (nicotinamide adenine dinucleotide, reduced form) [35] was placed in a shaking incubator at 35 °C for 18 h. After incubation, a ten-fold serial dilution ranging from 10^8^ to 1 CFU/mL was prepared in 0.9% NaCl by using a densitometer (starting at McFarland 0.5, measured with a densitometer, BioMérieux Marcy-l’Étoile, France). On each dilution step, a volume of 1 mL was used for DNA isolation with QIAGEN GmbH DNeasy Blood & Tissue Kit and was subsequently tested with the LAMP assay. For microbiological cell counts, a volume of 100 µL from each dilution step was spread on a chocolate agar plate with a hockey stick, followed by incubation at 35 °C for 24 h. Bacterial colonies were counted on chocolate agar plates (10 to 150 colonies) and the results were expressed in CFU/mL.

### 2.9. Investigation of Spiked Bronchoalveolar Lavage Fluid

For the LAMP assay validation, spiking experiments were performed under simulated conditions. As a suitable spiking matrix, bronchoalveolar lavage (BAL) fluid was taken from healthy pigs provided by the Field Station for Epidemiology in Bakum, Germany. To prove the absence of *G. parasuis* in the bronchoalveolar lavage fluid, samples were tested with the LAMP assay as well as with a conventional PCR assay, which was proved to be well suited in a previous study [24]. Moreover, a poly 16S rRNA gene PCR was performed to verify the presence of a bacterial DNA background. Furthermore, to determine the limit of detection in these samples, a ten-fold serial dilution of *G. parasuis* cells in 0.9% NaCl starting at 10^8^ (McFarland 0.5) up to 10^1^ CFU/mL was prepared as previously described. On each dilution step, 100 µL were spiked into 900 µL of the BAL fluid, while another 100 µL were spread on chocolate agar and incubated at 35 °C for 24 h to estimate the CFU/mL as described above. A volume of 100 µL from the spiked BAL was then diluted in 900 µL NaCl and centrifuged and the pellet was used for DNA isolation with a Qiagen Blood & Tissue kit. Five independent experiments were performed.

The agreement between the methods was calculated as a percentage of equal results, with upper and lower binomial confidence intervals calculated in accordance with the Clopper–Pearson method [40].

## 3. Results

### 3.1. Assay Optimisation

Two different target genes, 16S rDNA and *infB,* were compared in this study. The primer set targeting the 16S ribosomal RNA gene showed cross-reaction (positive amplification results in the LAMP assay) with *Mannheimia haemolytica* isolates and was therefore excluded from the further course of this study (data not shown). One primer set targeting the *infB* gene, which was designed with the software Primer Explorer, showed cross-reaction with *Actinobacillus minor* strain CCUG 38923^T^ and was therefore excluded as well. However, the second primer set (designed by LAMP design) targeting the *infB* gene resulted in positive amplifications for all tested *G. parasuis* isolates, but not for the other closely related bacterial strains and isolates included in the study. Due to its ability to differentiate between the bacterial species, this primer set was used for all further experiments. In addition, a temperature gradient was tested between 63 and 69 °C and the fastest amplification time was achieved with 66 °C (08:55 min at a DNA concentration of 0.1 ng/µL).

### 3.2. Analytical Sensitivity

Runs (*n* = 3) with concentrated primers in the master mix (for concentrations see Table 3) showed a detection limit of 10 fg/µL DNA with a mean detection time between 6:50 and 27:50 min (Table 4). In contrast, when using the standard concentrated primer mix, one order of magnitude was lost in the determination of the detection limit, and the lowest concentration that still led to amplification of the targets was 0.1 pg/µL DNA of the test strain with mean detection times between 10:25 and 19:15 min (Table 4). An example of an analytical sensitivity run in the Genie Explorer program is presented in Figure 2.

**Figure 4:**
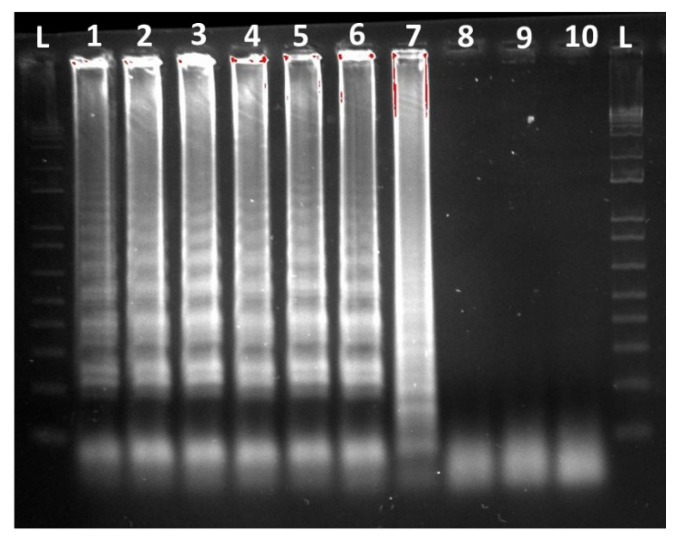
Analysis of the LAMP amplification products using gel electrophoresis. L lanes: 1 kb Plus DNA ladder. Lanes 1–8: amplification products from strain *G.* (*H.) parasuis* DSM 21448 starting at concentrations of 10 ng/µL up to 1 fg/µL. Lane 9: DNA from *Actinobacillus minor* CCUG 38923^T^. Lane 10: no template control.

In addition, an assay based on a colorimetric detection reaction was tested. The detection of a color change with the naked eye using a colorimetric master mix and amplification in the heating block resulted in a detection limit of 10 fg/µL, which is the same result as obtained with the Genie^®^ II assay (Figure 3). Even with the colorimetric detection, all negative controls remained visibly negative (shown in pink (samples 8–10)), while the positive samples turned dark yellow (samples 1–7). Gel electrophoresis confirmed results of the colorimetric test and showed a typical band pattern starting at the detection limit of 10 fg/µL DNA and for higher DNA concentrations (Figure 4).

Based on these results, a LAMP protocol with a temperature of 66 °C, a running time of 45 min, and the concentrated primers in the master mix was created and used for all further experiments.

### 3.3. Analytical Specificity

When using the optimized protocol, no cross-reactivity with any other Pasteurellaceae species (Actinobacillus minor, Actinobacillus indolicus, Actinobacillus porcinus, Actinobacillus arthritis, Actinobacillus pleuropneumoniae, Mannheimia haemolytica, Pasteurella multocida) or non-related species (Bordetella bronchiseptica) was observed, while all G. parasuis isolates showed a positive result. This corresponded to the inclusivity and exclusivity of 100%. According to the manufacturer of Genie^®^ II devices, a positive result is defined as the occurrence of an amplification product in combination with a peak in the melting curve (also designated by the manufacturer as annealing temperature) at the expected temperature. A melting temperature of 85.3 ± 0.6 °C was determined during the inclusivity tests.

### 3.4. Detection of G. parasuis in Spiked Bronchoalveolar Lavage Fluids and Limit of Detection

To demonstrate the suitability of the method for clinical samples, spiked BAL fluids, which had been previously identified as *G. parasuis*-free, were used. These samples all showed a bacterial background flora as determined by a poly 16S rDNA PCR assay [41]. In addition, spiked 0.9% NaCl samples were used. This enabled a comparison of two different matrices, which also differed in the presence and absence of a bacterial background flora. However, the CFU-based detection limit determined by two-fold serial dilution series of spiked NaCl samples was 3.5 × 10^2^ *G. parasuis* cells per mL NaCl, while spiked BAL fluids showed a mean detection limit of 2.58 × 10^2^ *G. parasuis* cells per mL (ranged between 2.1 × 10^2^ to 2.1 × 10^3^ cells/mL).

### 3.5. Detection of G. parasuis in Clinical Field Samples

Out of 36 organ swab samples, 11 were positive in the non-quantitative culture-based detection of *G. parasuis* (corresponding to a percentage of 30.6%), while 15 (41.6%) yielded positive results in the LAMP assay when undiluted DNA from the isolation kit was used (Table 5). This corresponded to a 77.1% agreement (95% confidence interval (CI): 59.9, 89.6) between the methods (Table 6). Using the conventional PCR assay as described earlier [24], 12 samples (33.3%) were PCR-positive. Thus, 91.4% (95% CI: 76.9, 98.2) and 85.7% (95% CI: 69.7, 95.2) of the PCR results were in agreement with the results of the LAMP with undiluted kit-extracted DNA or the results of the conventional PCR, respectively. In total, five of the samples tested negative with the culture-based method were positive in the LAMP assay, and in three of the cases, the LAMP result was confirmed by conventional PCR. In contrast, *G. parasuis* was detected in two swabs only by conventional microbiological examination. When comparing the two DNA extraction methods (kit and heat extraction, each with undiluted DNA; Table 5), the results only differed for two samples (swabs from the pericardium). Furthermore, genomic DNA was tested undiluted and diluted 1:10 to observe whether such an adjustment affected amplification. In the case of isolation with the kit, two samples gave a negative result after dilution compared to the undiluted samples, whereas dilution of DNA after heat extraction in most cases led only to faster detection times (Table 6).

## 4. Discussion

*Glaesserella (Haemophilus) parasuis* is an important pathogen in the swine industry and rapid detection is key to controlling Glässer’s disease and preventing possible outbreaks in herds. It is a sensitive fastidious pathogen dependent on nicotinamide adenine dinucleotide (NAD); therefore, its culture-dependent detection may not always be successful. In addition, culturing requires a relatively quick spread on growth media after sampling in order to maintain viability of the bacteria. Consequently, PCR-based methods provide many advantages in pathogen detection such as the possibility of storing samples for a longer period of time, quantification, or differentiation between morphologically indistinguishable species or genera.

In 2001, the first conventional PCR method for detecting *Glaesserella* (*Haemophilus) parasuis* was published [24]. For this assay, which targets a region of the 16S ribosomal DNA gene, difficulties in distinguishing between *G. parasuis* and *A. indolicus* were reported. Thus, the assay was further developed and a modified version of the PCR with better specificity but worse sensitivity results was published in 2007 . Currently, there are several PCR-based methods for detection of *G. parasuis*. However, these mostly require costly equipment that should be handled by experienced staff and is not easily transportable for field work. In 2000, a Japanese research team introduced a new detection method called loop-mediated isothermal amplification (LAMP) which amplifies DNA under isothermal conditions [23]. Some LAMP devices, such as the one used in this study, are portable devices that allow an ongoing run to be observed in real time and provide a melting temperature as a control. The device is equipped with a long-lasting battery and can be used off-grid directly in the field. Alternatively, a complete LAMP assay can be performed with a heat block only, allowing not only amplification, but also DNA heat extraction. The practical benefit of LAMP has already been confirmed by many previous studies [42,43].

In the assay shown here, different target genes were evaluated. One primer set targeting the *infB* gene showed a very good performance, while the other primer set for the same target gene did not provide reliable differentiation between *G. parasuis* and *Actinobacillus indolicus* isolates. A second alternative target gene encoding 16S rRNA was insufficiently specific to differentiate between *G. parasuis* and other closely related species, too. The inappropriateness of the 16S rRNA gene as the target gene was also described by Turni et al. (2010) [26]. The first approaches for detecting *G. parasuis* using the LAMP method have also been made. For example, Yang’s research group developed and evaluated LAMP-based detection of *G. parasuis* targeting the 16S rRNA gene [32], but the inclusion of important controls such as the testing of closely related species, e.g., *Actinobacillus indolicus* and others, which must give negative results in the assay, was missing. Another study [34] also focused on establishing a LAMP assay for detecting *G. parasuis*, but did not include these negative controls either. Therefore, an assay should be developed here that allows this differentiation and which can also be used for clinical samples. The advantages that should result from the assay are shown in Table 7.

The present assay correctly identified all 60 *G. parasuis* strains, including 14 different serotypes, and distinguished *G. parasuis* from all other tested species. Thus, it could be shown that the assay is 100% *G. parasuis*-specific. Particularly noteworthy is the successful differentiation of the assay between *G. parasuis* and *A. indolicus*, *A. minor*, *A. arthritis*, and *A. porcinus* due to the similarity in the target genome sequences and the previously reported problems in differentiation in other studies. This is of particular importance as these species also occur in pigs and colonize the upper respiratory tract.

During the establishment procedures, a temperature of 66 °C with a run time of 45 min provided the fastest amplification. The comparison of two primer concentration variants showed a ten-fold difference in sensitivity in favor of a concentrated primer mix that detected a minimum of 10 fg/µL. Yang et al. (2010) were able to detect positive *G*. *parasuis* only from 0.68 pg pathogen DNA per microliter with their LAMP method. In addition, our test showed a low detection limit (2.58 × 10^2^ cells) in spiked BAL fluids. The methods developed by Yang et al. [32], Chen et al. [33], and Zhang et al. [34] achieved detection limits of 8 CFU/mL, 10 CFU/mL, and five copies of DNA per tube, respectively. However, in none of these studies was BAL used as the spiking matrix and, therefore, no statement can be made concerning the suitability for direct detection from BAL fluids when using these methods. During the establishment of the LAMP assay, it was noticed that there were differences in the analytical sensitivity and the limit of detection between pure DNA detection and detection of cells in spiked NaCl or BAL fluids. These differences could be explained by the loss of DNA during isolation [44].

In this study, a second assay using a colorimetric master mix was tested and showed the same sensitivity for color-based detection of amplicons as in the case of amplification in a Genie^®^ II device. This assay was performed to present an alternative option when working in the field. It offers easy detection via the naked eye when using one device, a heating block or a water bath, for DNA extraction and amplification of samples under isothermal conditions. Similar colorimetric assays developed for other pathogens showed promising results [45,46]. Nevertheless, visual positivity might be secondarily confirmed in the laboratory by gel electrophoresis.

The main LAMP assay was validated by analyzing 36 swabs from different organs and the results thereof were compared to two gold standards—the bacterial culture-based detection (performed at the Field Station for Epidemiology, Bakum, Germany) and conventional PCR as described earlier [24]. However, slight deviations in the results were detected. Two cultural microbiologically positive samples were negative in LAMP and PCR. Since direct pathogen detection from culture material was otherwise always reliable, it could have been the case here that the swabs did not contain bacteria, e.g., if they were taken from a location different from the one used for culture detection. In other five cases, negative samples from cultural microbiological detection were found to be LAMP-positive. However, this can easily be explained by high sensitivity and particular nutrient dependence of the pathogen, which often makes cultivation very difficult [32]. This assumption was confirmed in three cases by the use of a second DNA-based detection method, conventional PCR, which also showed a positive result. A further possibility is, of course, that only DNA was detected here and that the pathogen was no longer viable and therefore culturing could not be successful. This difference between culture-based detection and DNA-based detection has been reported many times, e.g., for *Campylobacter* spp. [47], *Legionella* spp. [48], or *Streptococcus* spp. [49]. However, two samples were LAMP-positive, whereas PCR remained negative. The reason could be the presence of PCR inhibitors in the samples or a contaminating background which influences one method more than the other. The research group of Lee et al. came to similar conclusions, whereby LAMP was more tolerant to PCR inhibitors than conventional PCR and therefore yielded better results [50].

During the testing of field samples, we also focused on the comparison of two DNA extraction methods. The isolation with a commercial kit showed more positive results and also faster detection times. Nevertheless, heat extraction in the field can be used as an alternative DNA extraction method, e.g., for screening.

In conclusion, this improved LAMP assay targeting a region of the *infB* gene for detection of *G. parasuis* presents a highly sensitive, rapid, and easy-to-handle method that can be used as a good alternative to other traditional methods.

## Figures and Tables

**Table 1:** Numbers, sources, and serovars of bacterial strains and isolates used for inclusivity and exclusivity tests in this study (*G. parasuis* and non-*G. parasuis* isolates).

Species	*n*	Serovars	Source/Reference
*Glaesserella parasuis* DSM 21448^T^ type strain	1	ND	Leibniz-Institute DSMZ
*Glaesserella parasuis* field isolates	29	1,2,3,4,5,6,8,9,10,11,12,13,14,15	IVD GmbH; Institute collection [35,36]
*Glaesserella parasuis* field isolates	30	ND	
*Actinobacillus minor* CCUG 38923^T^ type strain	1		Culture Collection University of Gothenburg
*Actinobacillus indolicus* CCUG 39029^T^ type strain	1		Culture Collection University of Gothenburg
*Actinobacillus porcinus* CCUG 38924^T^ type strain	1		Culture Collection University of Gothenburg
*Actinobacillus arthritis* CCUG 24862^T^ type strain	1		Culture Collection University of Gothenburg
*Actinobacillus pleuropneumoniae* field isolates	26		Institute collection
*Mannheimia haemolytica* field isolates	11		Institute collection
*Pasteurella multocida* field isolates	20		Institute collection
*Bordetella bronchiseptica* field isolates	2		Institute collection

ND: not determined.

**Table 2:** Most suitable primer set used for the LAMP assay targeting the *infB* gene.

Primer	Sequence	Positions in *infB* (Accession No. DQ410886)	Length (bp)
F3	GCGTGAAGTGGCGTTAT	739	17
B3	GATAATGCGACGTGCTGA	1063	18
FIP (F1c+F2)	CAATCGCTTCTACAGAGCCTTGT- TTAGCAATATGGCGGAAGG	902, 822	23, 19
BIP (B1c+B2)	GCTCTGGCGTAGGTGGTATTAC- AAGGCAAGTACAATAGCGTT	954, 1026	22, 20
Loop F	TCACAATCACGTTCAATTCTGC	869	22
Loop B	AACCTTAGCGGCAGCG	988	16

**Table 3:** Different concentrations of the primer mix used.

Primers	Standard Concentration of the Primer Mix	Concentrated Primer Mix
F3, B3	0.2 µM	0.2 µM
FIP, BIP	0.8 µM	2 µM
Loop F, loop B	0.4 µM	1 µM

**Table 4:** Comparison of detection times using the standard and concentrated primer mixtures in a serial dilution (1 ng/µL–1 fg/µL) of DNA of the reference strain *G.* (*H.) parasuis* DSM 21448^T^ (*n* = 3).

**c (std. PM)**	**10 ng/µL**	**1 ng/µL**	**0.1 ng/µL**	**10 pg/µL**	**1 pg/µL**	**0.1 pg/µL**	**10 fg/µL**	**1 fg/µL**	**NTC**
M Dt (mm:ss)	10:25	11:30	12:55	14:40	16:55	19:50	-	-	-
SD Dt (mm:ss)	00:09	00:26	00:43	00:57	00:57	02:51	-	-	-
M At (°C)	85.83	85.83	85.97	85.93	85.90	85.97	-	-	-
**c (conc. PM)**	**10 ng/µL**	**1 ng/µL**	**0.1 ng/µL**	**10 pg/µL**	**1 pg/µL**	**0.1 pg/µL**	**10 fg/µL**	**1 fg/µL**	**NTC**
M Dt (mm:ss)	06:50	07:50	08:50	10:05	12:05	16:25	27:50	-	-
SD Dt (mm:ss)	00:09	00:09	00:09	00:09	00:09	01:40	11:25	-	-
M At (°C)	85.47	85.47	85.53	85.57	85.47	85.50	85.60	-	-

c = DNA concentration; std. PM = primer mix with standard primer concentration; conc. PM = concentrated primer mix; M = mean of all amplification times and annealing temperatures (*n* = 3); SD = standard deviation; Dt = detection time; At = annealing temperature (melting temperature); NTC = no template control; - = no positive signal.

**Table 5:** Results of 36 swab samples from different organs of pigs tested with the LAMP assay and a conventional PCR assay including a comparison of two different DNA extraction methods and diluted versus undiluted samples.

			DNA Isolation with KIT		HEAT-Extracted DNA		Results Summarized	
			Undiluted	Diluted 1:10	Undiluted	Diluted 1:10		
No.	Organ	MI	Dt (mm:ss)	Dt (mm:ss)	Dt (mm:ss)	Dt (mm:ss)	LAMP	PCR
1	pericardium	-	-	-	-	-	-	-
2	tonsils	-	-	-	-	-	-	-
3	tonsils	-	-	-	-	-	-	-
4	tonsils	-	-	-	-	-	-	-
5	tonsils	-	-	-	-	-	-	-
6	pericardium	-	23:15	-	-	-	+/-	-
7	pericardium	-	27:00	-	38:00	28:15	+	+
8	pericardium	-	-	-	-	-	-	-
9	pericardium	-	-	-	-	-	-	-
10	pericardium	-	-	-	-	-	-	-
11	pericardium	-	13:15	12:15	42:45	27:15	+	+
12	bronchi	+	14:30	15:15	35:45	13:30	+	+
13	bronchi	+	18:15	12:30	41:30	16:45	+	+
14	bronchi	+	13:30	13:15	35:45	12:15	+	+
15	bronchi	+	18:15	-	19:45	20:45	+	+
16	joint	+	14:15	10:45	41:45	23:00	+	+
17	serous membrane	-	14:15	14:30	44:00	28:00	+	+
18	brain	-	-	-	-	-	-	-
19	pericardium	+	19:45	14:00	24:45	14:45	+	+
21	bronchi	+	-	-	-	-	-	-
22	serous membrane	+	11:00	12:00	13:00	14:30	+	+
23	lung	+	-	-	-	-	-	-
24	pericardium	+	11:30	12:45	21:15	27:30	+	+
25	pericardium	+	14:00	15:45	-	-	+/-	+
26	pericardium	-	-	ND	ND	ND	-	-
27	bronchi	-	-	ND	ND	ND	-	-
28	serous membrane	-	-	ND	ND	ND	-	-
29	pericardium	-	-	ND	ND	ND	-	-
30	pericardium	-	-	ND	ND	ND	-	-
31	pericardium	-	18:15	ND	ND	ND	-	-
32	pericardium	-	-	ND	ND	ND	-	-
33	serous membrane	-	-	ND	ND	ND	-	-
34	pericardium	-	38:15	ND	ND	ND	-	-
35	pericardium	-	-	ND	ND	ND	-	-
36	pericardium	-	-	ND	ND	ND	-	-

SD (±) = standard deviation; Dt = detection time; MI = culture-based investigation; ND = not done; - = tested negative; + = tested positive, +/- = only one DNA extraction method is positive.

**Table 6:** Agreement of results between different methods for the detection of *G. parasuis* in clinical samples.

Detection Method Used		Agreement of Results in %	95% Confidence Intervals	
Variable 1	Variable 2		Lower	Upper
Culture-based detection	LAMP kit-extracted DNA, undiluted	77.1	59.9	89.6
Culture-based detection	LAMP, heat-extracted DNA, undiluted	75.0	53.3	90.2
Conventional PCR	LAMP kit-extracted DNA, undiluted	91.4	75.9	98.2
Conventional PCR	LAMP, heat-extracted DNA, undiluted	95.8	78.9	99.9
LAMP kit-extracted DNA, undiluted	LAMP kit-extracted DNA, diluted 1:10	87.5	67.6	97.3
LAMP, heat-extracted DNA, undiluted	LAMP kit-extracted DNA, diluted 1:10	100	85.8	100
LAMP kit-extracted DNA, undiluted	LAMP, heat-extracted DNA, undiluted	91.7	73.0	99.0
Culture-based detection	Conventional PCR	85.7	69.7	95.2

**Table 7:** The advantages of the developed LAMP assay.

Parameter	Availability for This Assay
Primer concentration	Yes
Amplification temperature	Yes
Inclusion of the fifth and sixth (loop) primers	Yes
Threshold	Yes
Real-time	Yes
Inclusion of closely related species in the determination of analytical specificity	*Actinobacillus minor, Actinobacillus indolicus, Actinobacillus porcinus, Actinobacillus arthritis* (among others)
Analytical sensitivity	10 fg/µL DNA
Analytical specificity	Inclusivity and exclusivity of 100%
Comparison of target genes	Yes
Comparison of DNA extraction methods	Yes
Detection in clinical field samples	Yes
Transportability to field	Yes
Reproducibility of the assay	Yes

## Data Availability

The data presented in this study are available within this article or in the supplementary material (Table S1).

## Supplementary Materials

The following are available online at https://www.mdpi.com/2076-2607/9/1/41/s1, Table S1: Sequences of all the designed primer sets.

## References

[1] Dickerman A., Bandara A.B., Inzana T.J. (2020). Phylogenomic analysis of *Haemophilus parasuis* and proposed reclassification to *Glaesserella parasuis*, gen. nov., comb. nov. Int. J. Syst. Evol. Microbiol..

[2] Kirkwood R., Rawluk S., Cegielski A., Otto A.J. (2001). Effect of pig age and autogenous sow vaccination on nasal mucosal colonization of pigs by *Haemophilus parasuis*. Swine Health Prod..

[3] Liu S., Li W., Wang Y., Gu C.-Q., Liu X., Charreyre C., Fan S., He Q. (2017). Coinfection with *Haemophilus parasuis* serovar 4 increases the virulence of porcine circovirus type 2 in piglets. Virol. J..

[4] Nedbalcová K., Šatrán P., Jaglic Z., Ondriasova R., Kucerova Z. (2006). *Haemophilus parasuis* and Glässer’s disease in pigs: A review. Vet. Med..

[5] Solano-Aguilar G.I., Pijoan C., Rapp-Gabrielson V., Collins J., Carvalho L.F., Winkelman N. (1999). Protective role of maternal antibodies against *Haemophilus parasuis* infection. Am. J. Vet. Res..

[6] Little T.W. (1970). Haemophilus infection in pigs. Vet. Rec..

[7] Peet R.L., Fry J., Lloyd J., Henderson J., Curran J., Moir D. (1983). *Haemophilus parasuis* septicaemia in pigs. Aust. Vet. J..

[8] Olvera À., Cerdag-Cuellar M., Mentaberre G., Casas-Díaz E., Lavín S., Marco I., Aragon V. (2007). First isolation of *Haemophilus parasuis* and other NAD-dependent Pasteurellaceae of swine from European wild boars. Vet. Microbiol..

[9] Reiner G., Fresen C., Bronnert S., Haack I., Willems H. (2010). Prevalence of *Haemophilus parasuis* infection in hunted wild boars (*Sus scrofa*) in Germany. Eur. J. Wildl. Res..

[10] Kielstein P., Rapp-Gabrielson V.J. (1992). Designation of 15 serovars of *Haemophilus parasuis* on the basis of immunodiffusion using heat-stable antigen extracts. J. Clin. Microbiol..

[11] Ma L., Wang L., Chu Y., Li X., Cui Y., Chen S., Zhou J., Li C., Lu Z., Liu J. (2016). Characterization of Chinese *Haemophilus parasuis* Isolates by Traditional Serotyping and Molecular Serotyping Methods. PLoS ONE.

[12] Pires Espíndola J., Balbinott N., Trevisan Gressler L., Machado G., Silene Klein C., Rebelatto R., Gutiérrez Martín C.B., Kreutz L.C., Schryvers A.B., Frandoloso R. (2019). Molecular serotyping of clinical strains of *Haemophilus* (*Glaesserella*) *parasuis* brings new insights regarding Glässer’s disease outbreaks in Brazil. PeerJ.

[13] Rapp-Gabrielson V.J., Gabrielson D.A.A. (1992). Prevalence of *Haemophilus parasuis* serovars among isolates from swine. Am. J. Vet. Res..

[14] Tadjine M., Mittal K.R., Bourdon S., Gottschalk M. (2004). Development of a New Serological Test for Serotyping *Haemophilus parasuis* Isolates and Determination of Their Prevalence in North America. J. Clin. Microbiol..

[15] Luppi A., Bonilauri P., Dottori M., Iodice G., Gherpelli Y., Merialdi G., Maioli G., Martelli P. (2013). *Haemophilus parasuis* Serovars Isolated from Pathological Samples in Northern Italy. Transbound. Emerg. Dis..

[16] Angen Ø., Svensmark B., Mittal K.R. (2004). Serological characterization of Danish *Haemophilus parasuis* isolates. Vet. Microbiol..

[17] Jia A., Zhou R., Fan H., Yang K., Zhang J., Xu Y., Wang G., Liao M. (2017). Development of Serotype-Specific PCR Assays for Typing of *Haemophilus parasuis* Isolates Circulating in Southern China. J. Clin. Microbiol..

[18] Cai X., Chen H., Blackall P.J., Yin Z., Wang L., Liu Z., Jin M. (2005). Serological characterization of *Haemophilus parasuis* isolates from China. Vet. Microbiol..

[19] Lin W.-H., Shih H.-C., Lin C.-F., Yang C.-Y., Chang Y.-F., Lin C.-N., Chiou M.-T. (2018). Molecular serotyping of *Haemophilus parasuis* isolated from diseased pigs and the relationship between serovars and pathological patterns in Taiwan. PeerJ.

[20] Amano H., Shibata M., Kajio N., Morozumi T. (1994). Pathologic Observations of Pigs Intranasally Inoculated with Serovar 1, 4 and 5 of *Haemophilus parasuis* Using Immunoperoxidase Method. J. Vet. Med. Sci..

[21] Segalés J., Domingo M., Solano G.I., Pijoan C. (1997). Immunohistochemical Detection of *Haemophilus Parasuis* Serovar 5 in Formalin-Fixed, Paraffin-Embedded Tissues of Experimentally Infected Swine. J. Vet. Diagn. Investig..

[22] Rafiee M., Blackall P.J. (2000). Establishment, validation and use of the Kielstein-Rapp-Gabrielson serotyping scheme for *Haemophilus parasuis*. Aust. Vet. J..

[23] Angen Ø., Oliveira S., Ahrens P., Svensmark B., Leser T.D. (2007). Development of an improved species specific PCR test for detection of Haemophilus parasuis. Vet. Microbiol..

[24] Oliveira S., Galina L., Pijoan C. (2001). Development of a PCR test to diagnose Haemophilus parasuis infections. J. Vet. Diagn. Investig..

[25] Howell K.J., Weinert L.A., Langford P., Rycroft A.N., Wren B.W., Maskell D.J., Tucker A.W., Peters S.E., Wang J., Hernandez-Garcia J. (2017). “Pathotyping” Multiplex PCR Assay for *Haemophilus parasuis*: A Tool for Prediction of Virulence. J. Clin. Microbiol..

[26] Turni C., Pyke M., Blackall P.J. (2010). Validation of a real-time PCR for *Haemophilus parasuis*. J. Appl. Microbiol..

[27] Notomi T., Okayama H., Masubuchai H., Yonekawa T., Watanabe K., Amino N., Hase T. (2000). Loop-mediated isothermal amplification (LAMP) of DNA. Nucleic Acids Res..

[28] Li Y., Fan P., Zhou S., Zhang L. (2017). Loop-mediated isothermal amplification (LAMP): A novel rapid detection platform for pathogens. Microb. Pathog..

[29] Domesle K.J., Yang Q., Hammack T.S., Ge B. (2018). Validation of a *Salmonella* loop-mediated isothermal amplification assay in animal food. Int. J. Food Microbiol..

[30] Da Silva S.J.R., Paiva M.H.S., Guedes D.R.D., Krokovsky L., De Melo F.L., Da Silva M.A.L., Da Silva A., Ayres C.F.J., Pena L.J. (2019). Development and Validation of Reverse Transcription Loop-Mediated Isothermal Amplification (RT-LAMP) for Rapid Detection of ZIKV in Mosquito Samples from Brazil. Sci. Rep..

[31] Zhang Y., Odiwuor N., Xiong J., Sun L., Nyaruaba R.O., Wei H., Tanner N.A. (2020). Rapid Molecular Detection of SARS-CoV-2 (COVID-19) Virus RNA Using Colorimetric LAMP. medRxiv.

[32] Wang Y., Fang Y., Liu Y., Chen P., Li W., Liu S., Zou H., He Q. (2010). Development and evaluation of loop-mediated isothermal amplification for rapid detection of *Haemophilus parasuis*. FEMS Microbiol. Lett..

[33] Chen H.-T., Chu Y.-F., Liu Y.-S., Zhang J., Lu Z. (2010). Loop-mediated isothermal amplification for the rapid detection of *Haemophilus parasuis*. FEMS Immunol. Med. Microbiol..

[34] Zhang J.-M., Shen H.-Y., Liao M., Ren T., Guo L.-L., Xu C.-G., Feng S.-X., Fan H.-Y., Li J.-Y., Chen J.-D. (2012). Detection of Haemophilus parasuis isolates from South China by loop-mediated isothermal amplification and isolate characterisation. Onderstepoort J. Vet. Res..

[35] Prüller S., Turni C., Blackall P.J., Beyerbach M., Klein G., Kreienbrock L., Strutzberg-Minder K., Kaspar H., Meemken D., Kehrenberg C. (2017). Towards a Standardized Method for Broth Microdilution Susceptibility Testing of *Haemophilus parasuis*. J. Clin. Microbiol..

[36] Brogden S., Pavlović A., Tegeler R., Kaspar H., De Vaan N., Kehrenberg C. (2018). Antimicrobial susceptibility of *Haemophilus parasuis* isolates from Germany by use of a proposed standard method for harmonized testing. Vet. Microbiol..

[37] Bisping W., Amtsberg G. (1988). Colour Atlas for the Diagnosis of Bacterial Pathogens in Animals.

[38] Sange M.D., Becker A., Hassan A.A., Bülte M., Ganter M., Siebert U., Abdulmawjood A. (2019). Development and validation of a loop-mediated isothermal amplification assay—A rapid and sensitive detection tool for *Mycobacterium avium* subsp. *paratuberculosis* in small ruminants. J. Appl. Microbiol..

[39] AOAC (2016). Guidelines for Standard Method Performance Requirements.

[40] SAS® Institute Inc (2013). User’s Guide (Release 9.4).

[41] Koukos G., Papadopoulos C., Tsalikis L., Sakellari D., Arsenakis M., Konstantinidis A. (2015). Prevalence of Antibiotic Resistance Genes in Subjects with Successful and Failing Dental Implants. A Pilot Study. Open Dent. J..

[42] Bath C., Scott M., Sharma P.M., Gurung R.B., Phuentshok Y., Pefanis S., Colling A., Balasubramanian N.S., Firestone S.M., Ungvanijban S. (2020). Further development of a reverse-transcription loop-mediated isothermal amplification (RT-LAMP) assay for the detection of foot-and-mouth disease virus and validation in the field with use of an internal positive control. Transbound. Emerg. Dis..

[43] Best N., Rawlin G., Suter R., Rodoni B., Beddoe T. (2019). Optimization of a Loop Mediated Isothermal Amplification (LAMP) Assay for In-Field Detection of *Dichelobacter nodosus* with aprV2 (VDN LAMP) in Victorian Sheep Flocks. Front. Vet. Sci..

[44] De Kok J.B., Hendriks J.C.M., Van Solinge W.W., Willems H.L., Mensink E.J., Swinkels D.W. (1998). Use of Real-Time Quantitative PCR to Compare DNA Isolation Methods. Clin. Chem..

[45] Lai M.-Y., Ooi C.-H., Jaimin J.J., Lau Y.-L. (2020). Evaluation of WarmStart Colorimetric Loop-Mediated Isothermal Amplification Assay for Diagnosis of Malaria. Am. J. Trop. Med. Hyg..

[46] Verma G., Sharma S., Raigond B., Pathania S., Naga K., Chakrabarti S.K. (2019). Development and application of fluorescent loop mediated isothermal amplification technique to detect *Phytophthora infestans* from potato tubers targeting ITS-1 region. 3 Biotech.

[47] Seinige D., Von Köckritz-Blickwede M., Krischek C., Klein G., Kehrenberg C. (2014). Influencing Factors and Applicability of the Viability EMA-qPCR for a Detection and Quantification of *Campylobacter* Cells from Water Samples. PLoS ONE.

[48] Lund V., Fonahn W., Pettersen J.E., Caugant D.A., Ask E., Nysaeter Å. (2014). Detection of *Legionella* by cultivation and quantitative real-time polymerase chain reaction in biological waste water treatment plants in Norway. J. Water Health.

[49] Grønbaek L.M., Angen Ø., Vigre H., Olsen S.N. (2006). Evaluation of a nested PCR test and bacterial culture of swabs from the nasal passages and from abscesses in relation to diagnosis of *Streptococcus equi* infection (strangles). Equine Vet. J..

[50] Lee S., Khoo V.S.L., Medriano C.A.D., Lee T., Park S.-Y., Bae S. (2019). Rapid and in-situ detection of fecal indicator bacteria in water using simple DNA extraction and portable loop-mediated isothermal amplification (LAMP) PCR methods. Water Res..

