# Supplementary material for: Development and Validation of a Loop-Mediated Isothermal Amplification (LAMP) Assay for Rapid Detection of Glaesserella (Haemophilus) parasuis
Source: Microorganisms. 2020 Dec 25;9(1):41. doi: 10.3390/microorganisms9010041 (PMC7823361; doi:10.3390/microorganisms9010041)
Supplement: Supplementary file 1 [file microorganisms-09-00041-s001.pdf]

Supplementary data

Table S1: Primer set 1 targeting the 16S ribosomal RNA gene (accession number AB078973.1) designed with PrimerExplorer V4, primer set 2 targeting the *infB* gene (accession number DQ410886) designed with PrimerExplorer V4 and primer set 3 (chosen set) targeting the *infB* gene (accession number DQ410886) designed with LAMP Designer.

	**Primer set 1**	**Primer set 2**	**Primer set 3**
**F3**	GCCCTTACGAGTAGGGCTAC	GAAGTGGCGTTATACCGT	GCGTGAAGTGGCGTTAT
**B3**	GCCCCCCTTCGTTAAGC	ATACCACCTACGCCAGAG	GATAATGCGACGTGCTGA
**FIP**	GTTGCAGACTCCAATCCGGACTCATACAGAGGGTGACGAAGC	CCTTCCGCCATATTGCTAAACATATCAAGGTAAATTCCGTGATGTG	CAATCGCTTCTACAGAGCCTTGTTTAGCAATATGGCGGAAGG
**BIP**	ATCGCGAATCAGAATGTCGCGGACCCACTCCATGGTGTGA	TGAACGTGATTGTGAAAGCAGACTTCTGCCGTTGAAAGCT	GCTCTGGCGTAGGTGGTATTACAAGGCAAGTACAATAGCGTT
**LF**	AGATTCACTCCACCTCGCG	CTTTTTGTTGGCGAGCCAATTT	TCACAATCACGTTCAATTCTGC
**LB**	TGAATACGTTCCCGGGCCTTG	CGTACAAGGCTCTGTAGAAGCG	AACCTTAGCGGCAGCG
